# Screening of osteoporosis and sarcopenia in individuals aged 50 years and older at different altitudes in Yunnan province: Protocol of a longitudinal cohort study

**DOI:** 10.3389/fendo.2022.1010102

**Published:** 2022-11-14

**Authors:** Xingli Liu, Cunwen Ma, Shiping Wang, Zhengrong Liang, Juntao Yang, Jun Zhou, Yi Shu, Zhengying He, Jilong Zong, Lizhi Wu, Peiqian Peng, Yi Su, Meng Gao, Kaiming Shen, Hong Zhao, Jilu Ruan, Shaoxuan Ji, Yunhui Yang, Taisong Tang, Zongfa Yang, Guangyin Luo, Meng Zeng, Weiwan Zhang, Bo He, Xiaoguang Cheng, Gang Wang, Ling Wang, Liang Lyu

**Affiliations:** ^1^ Faculty of Life science and Technology, Kunming University of Science and Technology, Kunming, China; ^2^ Medical School, Kunming University of Science and Technology, Kunming, China; ^3^ Department of Radiology, The First People’s Hospital of Yunnan Province, Kunming, Yunnan, China; ^4^ Department of Radiology, The Affiliated Hospital of Kunming University of Science and Technology, Kunming, China; ^5^ Department of Radiology, The People’s Hospital of Wenshan Prefecture, Wenshan, China; ^6^ Department of Radiology, Anning First people’s Hospital, Kunming University of Science and Technology, Anning, China; ^7^ Department of Radiology, Qujing Second People’s Hospital of Yunnan Province, Qujing, China; ^8^ Department of Radiology, Dali Bai Autonomous Prefecture People’s Hospital, Dali, China; ^9^ Department of Radiology, Xishuangbanna Dai Autonomous Prefecture People’s Hospital, Jinghong, China; ^10^ Department of Radiology, Southern Central Hospital of Yunnan Province, Honghe, China; ^11^ Department of Radiology, Diqing Tibetan Autonomous Prefecture People’s Hospital, Xianggelila, China; ^12^ Department of Radiology, The First People’s Hospital of Zhaotong, Zhaotong, China; ^13^ Department of Radiology, Hekou People’s Hospital, Honghe, China; ^14^ Department of Radiology, Nujiang People’s Hospital, Nujiang, China; ^15^ Key Laboratory of Molecular Epidemiology of Hunan Province, School of Medicine, Hunan Normal University, Changsha, China; ^16^ Department of Radiology, The First Affiliated Hospital of Kunming Medical University, Kunming, China; ^17^ Department of Radiology, Beijing Jishuitan Hospital, Beijing, China

**Keywords:** osteoporosis, sarcopenia, elderly, altitudes, protocol

## Abstract

**Introduction:**

Musculoskeletal system gradually degenerates with aging, and a hypoxia environment at a high altitude may accelerate this process. However, the comprehensive effects of high-altitude environments on bones and muscles remain unclear. This study aims to compare the differences in bones and muscles at different altitudes, and to explore the mechanism and influencing factors of the high-altitude environment on the skeletal muscle system.

**Methods:**

This is a prospective, multicenter, cohort study, which will recruit a total of 4000 participants over 50 years from 12 research centers with different altitudes (50m~3500m). The study will consist of a baseline assessment and a 5-year follow-up. Participants will undergo assessments of demographic information, anthropomorphic measures, self-reported questionnaires, handgrip muscle strength assessment (HGS), short physical performance battery (SPPB), blood sample analysis, and imaging assessments (QCT and/or DXA, US) within a time frame of 3 days after inclusion. A 5-year follow-up will be conducted to evaluate the changes in muscle size, density, and fat infiltration in different muscles; the muscle function impairment; the decrease in BMD; and the osteoporotic fracture incidence. Statistical analyses will be used to compare the research results between different altitudes. Multiple linear, logistic regression and classification tree analyses will be conducted to calculate the effects of various factors (e.g., altitude, age, and physical activity) on the skeletal muscle system in a high-altitude environment. Finally, a provisional cut-off point for the diagnosis of sarcopenia in adults at different altitudes will be calculated.

**Ethics and dissemination:**

The study has been approved by the institutional research ethics committee of each study center (main center number: KHLL2021-KY056). Results will be disseminated through scientific conferences and peer-reviewed publications, as well as meetings with stakeholders.

**Clinical Trial registration number:**

http://www.chictr.org.cn/index.aspx, identifier ChiCTR2100052153.

## Introduction

Osteoporosis has become a public health concern with the rate of aging in the global population increasing (1). Identifying the risk factors for osteoporosis could help prevent the condition’s development. Environmental factors (such as altitude, sunlight, and temperature) have been proposed to influence bone mineral density (2). Basu M et al. found proximal phalanx bone impairment in healthy males who migrated from an altitude of 3542m to an extreme altitude (5400–6700m) in India where they stayed for 4 months (3). A Chinese study reported that people living in Tibet have lower spine bone mass compared with people who live at low altitudes (4). An animal experiment also showed that bone mass was significantly and negatively affected by exposure to a high-altitude environment. With the increase in altitude (1850~5450m), negative changes in the morphometric and geometric properties of the femur were observed (5). The loss of bone mass and/or decrease in bone density is the main manifestation of osteoporosis. As expected, several studies based on large sample sizes have found that highlanders have a higher risk of osteoporosis (6) and are more likely to suffer hip fractures (7–9). The above research results may be related to the mechanism that high altitude-induced hypoxia may stimulate the secretion of many hormones that have affected bone mineral metabolisms (3, 10). For example, activities of bone-specific alkaline phosphatase and 25(OH) vitamin D were both found to decrease significantly in the high-altitude area (3). Notably, the relationship between high altitude and bone status is still insufficient. Existing studies are also limited by their designs for certain people and/or small sample sizes. In particular, some studies only included narrow-altitude ranges (about 100~200 m), which restricts effective comparisons between distinct altitudes. This can sometimes lead to conflicting results (9, 11). A reasonable and wider range of elevation comparison design can contribute to explore the effect of hypoxia on bone and reveal the potential effective threshold. So far, no studies have been conducted in China on the prevalence of osteoporosis in the general population in high-altitude areas. More studies, especially longitudinal cohort studies, are essential to investigate the prevalence of osteoporosis and the potential risk factors for bone mass loss in the plateau area.

Hypoxia causes complex angio-adaptive and endocrine adaptations in skeletal muscle, resulting in the growth, stabilization, or regression of muscle capillaries as well as changes in blood biochemical markers (e.g., significant reductions in plasma leptin and homocysteine, insulin, and C-reactive protein) (12, 13). High-altitude hypoxic environments have been demonstrated to influence a person’s body composition (e.g., reductions in body weight, fat-free mass, fat mass, muscle mass, and/or body water) (14–17). Studies have been conducted on the relationship between altitude and muscle or body function, particularly in sports training. Altitude hypoxia training has become a popular means to increase endurance athletes’ performance for decades (18–22). What’s more, current research indicates that chronic intermittent hypoxic–hyperoxic periods exposure at rest is beneficial for older patients with cardiovascular and metabolic diseases or cognitive impairment to improve physical and cognitive performance and reduce cardiometabolic risk factors (23). Despite much research in this area to date, the results are highly controversial as intraindividual and interindividual variabilities (24, 25). More studies are needed to confirm and extend the evidence.

The loss of muscle mass, strength, and/or physical function is often referred to as sarcopenia, which is closely related to adverse clinical outcomes (15–17). Sarcopenia will have a major impact on the Asian aging population (26). The diagnosis of sarcopenia is also highly variable due to race, measurement methods, and living environments (27, 28). High-altitude hypoxia is considered to be able to facilitate sarcopenia and fat distribution (26, 29, 30). Chinese studies indicated that the incidences of sarcopenia in the high-altitude population (altitude>3500m and altitude at 2260m) were significantly higher than those in the plain population (26, 31). Specific cutoff values established according to altitudes for sarcopenia in the plateau populations seem more reasonable. However, very limited relevant studies are available. The assessment of muscle mass is a crucial element in diagnosing sarcopenia. Bioelectrical impedance analysis (BIA) or dual X-ray absorptiometry (DXA) were the most commonly used methods in the past. Nevertheless, the correlation of muscle mass with muscle strength, and more generically, with muscle function is low, and this discrepancy may be partially related to the presence of fatty infiltration (32, 33). The recent European Working Group on Sarcopenia in Older People recently replaced “low muscle mass” with “low muscle strength” as a primary determinant of sarcopenia (34) implying that muscle mass based on DXA and BIA may not be sufficient in the detection of sarcopenia and that methods with higher sensitivity in sarcopenia screening are warranted. Skeletal muscle area based on segmentation technology and muscle density measured by quantitative computed tomography (QCT) were considered to be more promising in the assessment of sarcopenia (35–37). However, the sarcopenia definition based on CT assessments of muscle size and density is lacking.

The musculoskeletal system operates as a finely coordinated unit, interconnected not only by mechanical aspects but also by humoral factors. Muscle seems to possess the “upper hand” in its relationship with bone (38). Muscle loading induces a range of biomechanical signals necessary for bone growth and remodeling. Also, osteoporosis or fractures will lead to muscle atrophy and muscle mass reduction (38). Indeed, several muscles and bone-derived hormones (e.g., leptin, insulin, GH/IGF-1, myostatin, FGF2, and sexual steroids) are under active investigation to better explain the complex cross-talk and discrete hormonal influences between muscle and bone (38). Therefore, simultaneous assessment of bone and muscle may help gain a more complete picture of disease prevention and treatment in a certain area.

High altitude is generally defined as an altitude higher than 1500m above sea level, which is further classified into three grades: high altitude (1500-3500m); ultra-high altitude (3500-5500m); and very high altitude (>5500 meters) (39). The main environmental stressor associated with high altitude is decreasing atmospheric oxygen pressure. Other environmental stressors include low temperatures, humidity, and increased ultraviolet radiation (40). The impact of altitude on the musculoskeletal system is diverse and contingent upon the altitude in question. Existing studies are limited by their designs for comparison between two altitudes (26, 41). As a result, studies at different altitudes cannot be directly compared. Multi-altitude control studies can comprehensively depict the changes in the musculoskeletal system at different altitudes.

As a province with an altitude fluctuation ranging from less than 100 meters to more than 3,000 meters, Yunnan province has a particular advantage in the study of the effects of altitude on osteoporosis and sarcopenia. This study is a multi-center cohort study in 12 regions where the altitude fluctuations range from 50 to 3500 meters. The primary aims of this study are listed as follows: Firstly, to compare the baseline prevalence of osteoporosis and sarcopenia in the over-50-year-old population at different altitudes and establish a provisional cut-off point based on QCT for the diagnosis of sarcopenia in adults according to altitude. Secondly, the bone and muscle characteristics of people at different altitudes are compared, such as bone density, muscle mass, muscle density, and biological indicators. Thirdly, follow up for 5 years and compare the incidence of adverse events (fall, osteoporosis fracture, death, etc) at different altitudes. The secondary study aims to explore the effect and mechanism of a high-altitude environment on the skeletal muscle system.

## Methods and analysis

This longitudinal cohort study aims to investigate the prevalence of osteoporosis and sarcopenia in Yunnan Province. Multicenter control at different altitudes can give insight into how altitude affects bone and muscle.

### Study design

**Figure 1:**
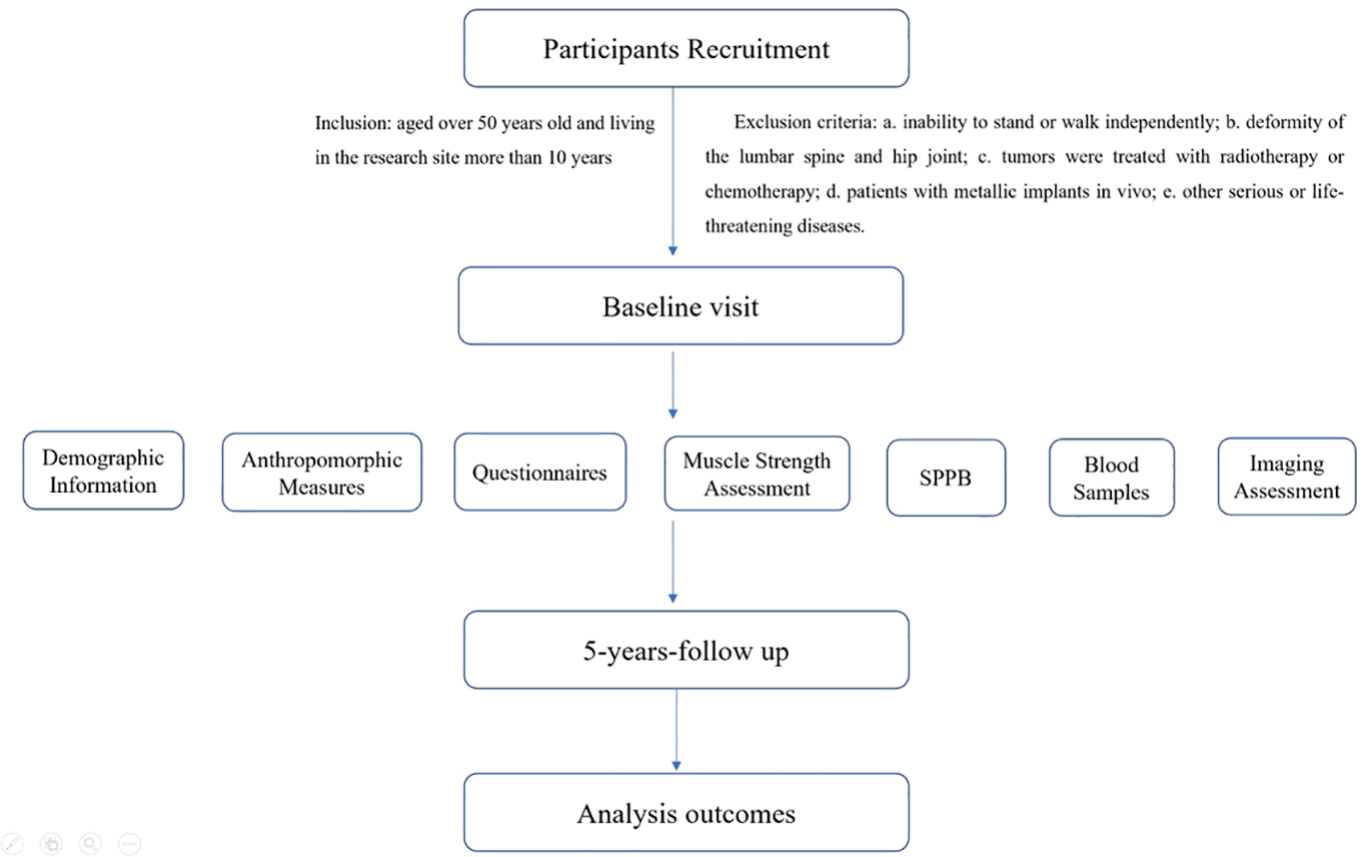
Flow chart of study design and procedures. SPPB, short physical performance battery. The analysis outcomes included: the changes in muscle size, density, and fat infiltration in different muscles, the decrease in BMD, the muscle function impairment, and the osteoporotic fracture incidence during a five-year follow-up period.

The sarcopenia and osteoporosis study in Yunnan province (SOY study, Trial registration number: ChiCTR2100052153) is a multicenter, prospective, cohort study. The study protocol consists of three main steps: recruitment, a baseline visit, and five years follow-up visit (**Figure1**).

### Recruitment strategy

**Figure 2:**
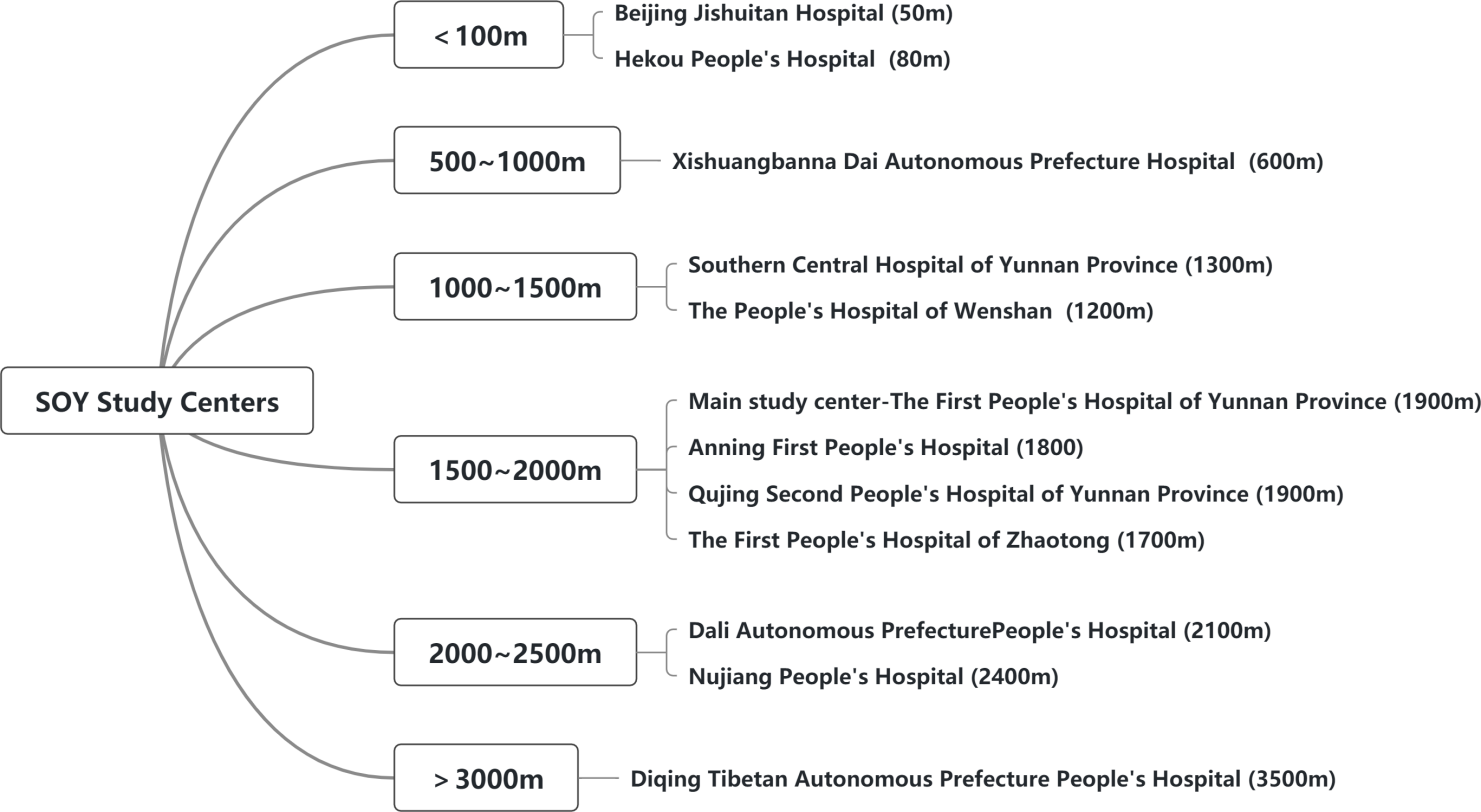
Recruitment centers and specific altitude.

The enrolment of potential subjects is assessed by investigators at each clinical center according to the inclusion and exclusion criteria. Each subject will sign an informed consent form before officially entering the study. The subjects of the SOY study are enrolled in 12 centers at different altitudes (**Figure 2**). The inclusion criteria are that participants should be aged over 50 years old and living at the research site for more than 10 years. Exclusion criteria were as follows: a. Inability to stand or walk independently; b. Deformity of the lumbar spine and hip joint; c. Tumors treated with radiotherapy or chemotherapy; d. Patients with metallic implants *in vivo*; e. Other serious or life-threatening diseases (e.g. severe stroke). Recruitment will start in August 2022 except for the main study center the First People’s Hospital of Yunnan Province in which recruitment started in April 2021. Baseline visits and follow-up details are shown in **Table 1**.

**Table 1:** Items and procedures of the study at baseline and each follow-up.

	Baseline	1 year ± 30 days	2 years ± 30 days	3 years ± 30 days	4 years ± 30 days	5 years ± 30 days
Informed consent	X	–	–	–	–	–
Questionnaire survey	X	X	X	X	X	X
Muscle strength assessment (HGS)	X	X	X	X	X	X
Short physical performance battery (SPPB)	X	X	X	X	X	X
QCT scan	X	X	X	X	X	X
DXA scan	X^*^	X^*^	X^*^	X^*^	–	X^*^
Ultrasonic	X^*^	X^*^	X^*^	X^*^	–	X^*^
Blood collection	X^*^	X^*^	X^*^	X^*^	X^*^	X^*^

X, an item that will be collected.

X^*^, item that will be selectively carried out in viable centers.

-, item that will not be collected.

Ethical approval for the cohort study is obtained from the ethics committee of each study center [Main study center-The First People’s Hospital of Yunnan Province No. KHLL2021-KY056]. The study is conducted by ethical principles according to the Declaration of Helsinki. Radiation safety and protection measures are strictly implemented throughout the study. Informed consent is obtained from each participant at the nearest participating imaging center.

### The estimation of sample size

The sample size necessary for this study is set at 4000. The overall prevalence of osteoporosis at the femoral neck in adults aged above 50 years was reported to be 16%, or even up to 30% in postmenopausal women (29, 42). The overall prevalence of sarcopenia was reported to range from 5.5% to 25.7% (30). Thus, the number of 4000 is set to get the estimated overall prevalence of osteoporosis to be within 5% of the prevalence in the real world and considering the attrition rate of about 10%. This number is also needed to detect a significant difference (at least 5%) in prevalence proportions between the highest level of altitude and the lowest one. This number is also needed to detect significant results from the prospective cohort (survival analysis) with a two-tailed level of significance of 5% and statistical power of 80% when a risk factor for osteoporotic fracture is assumed to exist in 20% of participants at baseline and to increase a background fracture risk of 6% by 50% during the 5-year follow-up period. The SPSS 26.0 software (IBM, Armonk, NY, United States) will be chosen. The level of significance desired for this study is α=0.05, with a power level of β=0.2.

### Baseline visit

The demographic information, anthropomorphic measures, questionnaires, muscle strength assessment, short physical performance battery (SPPB), collection of blood samples, and imaging assessments (QCT and/or DXA, US) are scheduled to be conducted on each participant.

Detailed demographic measures include age, biological age calculated based on somatic variables (43), gender, ethnicity, residence, education, occupational status, daily physical questionnaire (IPAQ), eating habits (e.g. tea intake, vegans, red meat/white meat intake/both), fall risk screening, fracture history (time/frequency/fracture site), alcohol intake, smoking history, medical history (calcium/vitamin D/hormone), menopausal age, fall history (time, frequency, location), disease history (rheumatoid arthritis/secondary osteoporosis).

Anthropomorphic measures consist of height, weight, waist, and maximum calf circumference. The SARC-F questionnaire will be used to predict potential persons with sarcopenia at risk for poor functional outcomes (described below **Table 2**).

**Table 2:** SARC-F questionnaire.

Component	Question	Score
Strength	How much difficulty do you have in lifting and carrying 10 pounds?	one = 0 Some = 1 A lot or unable = 2
Assistance in walking	How much difficulty do you have walking across a room?	None = 0 Some = 1 A lot, use aids, or unable = 2
Rise from a chair	How much difficulty do you have transferring from a chair or bed?	None = 0 Some = 1 A lot or unable without help = 2
Climb stairs	How much difficulty do you have climbing a flight of 10 stairs?	None = 0 Some = 1 A lot or unable = 2
Falls	How many times have you fallen in the past year?	None = 0 1-3 falls = 1 4 or more falls = 2

Muscle strength assessment with HGS. HGS of the dominant hand will be measured using a Jamar dynamometer (Jamar, Los Angeles, CA), two attempts with a 30-second interval between them were recorded in kilograms, and the maximum value will be chosen for further analysis.

A short physical performance battery (SPPB) includes a 4m gait speed (GS), a five-times repeated chair sit-to-stand (STS) and a balance test (semi-tandem, full-tandem, and single-leg stand time) will be recorded.

Blood sample collection is mainly for laboratory examination, such as hepatic and renal function, 25(OH)D, BGP, PTH, Calcium, biochemical markers of bone turnover, biochemical markers of bone metabolism, leptin, insulin, GH/IGF-1, myostatin, FGF2, and sex steroids.

**Figure 3:**
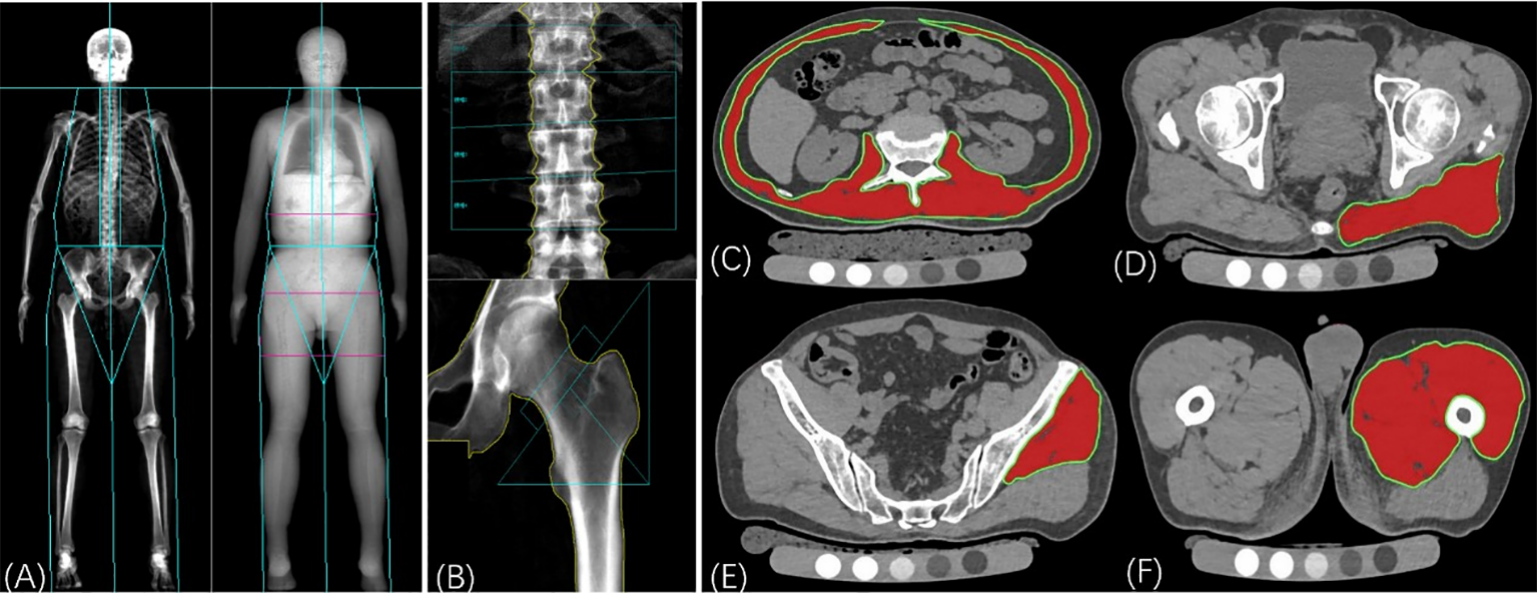
Dual-energy X-ray absorptiometry (DXA) and computed tomography (CT) assessments. **(A)**: the whole body DXA scan for body composition analysis, mainly for the evaluation of muscle and fat mass. **(B)**: Areal BMD was calculated by the DXA scan of the lumbar spine and left hip. **(C)**~**(F)**: Muscle assessments with QCT including muscle density, muscle area, and intermuscular fat area. **(C)**: Measurement of trunk muscle at mid-L2 level; **(D)**: Measurement of cross-sectional area and mean computed tomography values of the gluteus maximus muscle at the level of the greater trochanter of the femur; **(E)**: Measurement of the gluteus medius and minimus muscle at the third sacral level; **(F)**: Measurement of the middle thigh muscle level. The muscle region is represented by the area highlighted in red. The area of intramuscular fat infiltration is obtained by subtracting the area of red ROI from the area of green ROI with the threshold segmentation method.

Imaging assessments include abdomen quantitative computed tomography (QCT), dual-energy X-ray absorptiometry (DXA), and appendicular limb ultrasound. Evaluation indicators include muscle density, muscle size, intermuscular fat size, BMD (measured by QCT and/or DXA), and whole-body composition analysis. The trunk muscle, gluteus muscle, and appendicular limb muscle are the object muscles being evaluated. The BMD values (mg/cm^3^) of the L1 and L2 vertebral bodies are measured according to the QCT protocol (44) (**Figure 3**). The spinal vBMD was represented by the average vBMD value of L1–L2. In each subject, abdominal CT scans with a Mindways calibrated QCT acquisition phantom (Mindways Software Inc, Austin, TX, USA). For cross-calibration, a single European Spine Phantom (ESP-122) will be scanned at all centers before scanning the subjects.

Notably, QCT is a required item for all research centers.

### Follow-up visit

A 5-year follow-up will be conducted to evaluate the changes in muscle size, density, and fat infiltration in different muscles, the decrease of BMD, muscle function impairment, and osteoporotic fracture incidence. Details of the assessments of the follow-up visits are shown in **Table 1**.

### The diagnosis criteria of osteoporosis

The diagnostic criteria of osteoporosis for QCT, recommended by the International Society for Clinical Densitometry in 2007 (45) and the American College of Radiology in 2008 (46), are used to classify the subjects as normal if average vBMD >120 mg/cm^3^, osteopenia if vBMD between 120 and 80 mg/cm^3^, and osteoporosis if vBMD <80 mg/cm^3^.

### The diagnosis criteria of sarcopenia

According to the 2019 consensus update on sarcopenia diagnosis and treatment of the Asian working group for sarcopenia (AWGS 2019), the new diagnosis of sarcopenia was low muscle mass accompanied by low muscle strength or low physical performance. AWGS 2019 also defines persons with low muscle mass, low muscle strength, and low physical performance as having “severe sarcopenia” (30).

The specified cutoffs for each diagnostic component were as followed: low muscle strength was defined as handgrip strength < 28 kg for men and <18 kg for women; criteria for low physical performance were 6-m walk<1.0 m/s, short physical performance battery(SPPB) score ≤ 9, or 5-time chair stand test ≤ 12 seconds. Muscle mass assessment was retained the original cutoffs for height-adjusted: dual-energy X-ray absorptiometry<7.0 kg/m^2^ in men and <5.4 kg/m^2^ in women; and bioimpedance<7.0 kg/m^2^ in men and <5.7 kg/m^2^ in women.

### Data management

All data will be transferred to the First People’s Hospital of Yunnan Province for analysis and quality control. The study data will be collected and managed in a database created using Epidata 3.1. Through this database, all questionnaire contents can be digitized to prepare for further classification, comparison, and statistical analysis, such as activity, diet habits, medication history, etc. All muscle function evaluations, imaging scans, and measurements were performed according to the unified standards of the study. All investigators taking care of data collection will be trained before the project begins.

### Statistical analysis

Baseline cross-sectional analysis between sarcopenia/osteoporosis and altitude will be conducted, including the prevalence, imaging parameters, SPPB, and blood sample indexes of skeletal muscle. The 5-year follow-up data will focus on comparing the rate of skeletal muscle degradation at different altitudes and the incidence of adverse events such as falls and fractures during the follow-up period. One-way ANOVA will be used to compare the research results between different altitudes. Multiple linear and logistic regression analyses were conducted to calculate the effects of various factors (e.g., altitude, age, and physical activity) on the skeletal muscle system in a high-altitude environment. Cox proportional hazards models were used to calculate the strength of BMD, muscle density, and muscle mass to predict the risk of major osteoporotic fractures. Provisional cutoff points based on QCT were defined for the variables used to screen for sarcopenia or osteoporosis using the 20th percentile of their population distributions.

## Discussion

Osteoporosis and sarcopenia are highly prevalent in older adults. Studies have shown that osteoporosis and hip fractures are more common in high-altitude areas. However, the extent to which altitude affects bones, or how, is not yet clear. There are few studies on muscle at high altitudes, and the results are inconsistent at different study altitudes. So, it is necessary to make a systematic comparison at multiple altitudes. What’s more, the current diagnostic criteria for osteoporosis are relatively well-established, but there are still many uncertainties about the diagnostic criteria for sarcopenia. Many researchers are trying to propose more suitable diagnostic criteria for sarcopenia (47, 48). But so far, there is no specific diagnostic cut-off value for sarcopenia for residents at high altitudes.

To the best of our knowledge, this is the first multicenter cohort study with multiple altitude levels. The 5-year follow-up study design enables us to compare the changes of bone and muscle not only horizontally in time, but also longitudinally at different altitudes. This makes it possible to better describe the relationship between the skeletal muscle system and altitude environment, then put forward a more objective and reasonable explanation for the situation in which existing research results are inconsistent or even opposite due to different study altitudes (26, 41).

As far as we know, this is also the first study to assess bone and muscle at multiple altitudes simultaneously. Analysis of musculoskeletal interactions can provide valuable information about how the altitude environment affects the body.

In conclusion, this study will make an important contribution to the understanding of the health status of bone and muscle at different altitudes in Yunnan. The relationship between the musculoskeletal system and altitude can be more comprehensively discussed because of the availability of multiple altitude data sets. The study will provide essential data for developing individualized diagnostic criteria for sarcopenia in Yunnan and help to establish altitude-specific intervention and treatment strategies.

### Strengths and limitations of this study

The strengths of this study were listed as follows: Firstly, this is the first multicenter cohort study about bone and muscle characteristics of adults at different altitudes and would provide valuable reference data for adults aged over 50 years in plateau areas.

Secondly, the 5-year follow-up design provides longitudinal comparative data. Detailed changes and potential relationships in bone mass and muscle characteristics of older adults in high-altitude hypoxia environments can be dynamically recorded for further analysis. QCT and/or DXA measurements can provide more accurate assessment means of osteoporosis and sarcopenia diagnosis.

By the way, the limitations of this study are deserving of attention. Firstly, there is potential for high dropout rates of older adult participants due to physical decline or death. Secondly, external validation is lacking in this study.

## Ethics statement

The studies involving human participants were reviewed and approved by The First People’s Hospital of Yunnan Province KHLL2021-KY056. The patients/participants provided their written informed consent to participate in this study.

## Author contributions

GW, LW, LL: Conceptualization; Project administration; Funding acquisition; Supervision, Writing - Review & Editing. XL: Data Curation; Data analysis; Writing - Original Draft; Supervision, investigators training. CM, SW, ZL, JY, JuZ, YSh, ZH, JiZ, LZW, PP, MG, KS, HZ, JR, SJ, YY, TT, ZY, GL, MZ, WZ, XC, BH: Subject recruitment; Research data collection and recording. YS: Sample size calculation, statistical analysis. All authors contributed to the article and approved the submitted version.

## Funding

This work was supported by the national natural science foundation of China [grant number:81901718, 81771831]; Beijing hospitals authority youth program [grant number: qml20200402]; The Beijing hospitals authority clinical medicine development of special funding support [grant number: zylx202107]; Yunnan “ten thousand people plan” famous doctor special project [grant number: ynwr-my-2019-011] and The Clinical Medical Center Open Project of the first people’s hospital of Yunnan province [grant number:2022YJZX-LN10, 2022LCZXKF-HX06].

## Conflict of interest

The authors declare that the research was conducted in the absence of any commercial or financial relationships that could be construed as a potential conflict of interest.

## Publisher’s note

All claims expressed in this article are solely those of the authors and do not necessarily represent those of their affiliated organizations, or those of the publisher, the editors and the reviewers. Any product that may be evaluated in this article, or claim that may be made by its manufacturer, is not guaranteed or endorsed by the publisher.
